# Network Pharmacology and Bioinformatics Analyses Identify the Core Genes and Pyroptosis-related Mechanisms of *Nardostachys chinensis* for Atrial Fibrillation

**DOI:** 10.2174/0115734099259071231115072421

**Published:** 2024-01-03

**Authors:** Weiqi Xue, Yuan Luo, Weifeng He, Mengyuan Yan, Huanyi Zhao, Lijin Qing

**Affiliations:** 1 First School of Clinical Medicine, Guangzhou University of Chinese Medicine, Guangzhou, China;; 2 First Affiliated Hospital of Guangzhou University of Chinese Medicine, Guangzhou, China

**Keywords:** Atrial fibrillation, *Nardostachys chinensis*, pyroptosis, bioinformatic analysis, network pharmacology, immune cell infiltration

## Abstract

**Background:**

*Nardostachys chinensis* is an herbal medicine widely used in the treatment of atrial fibrillation (AF), but the mechanism is unclear.

**Objective:**

To explore the molecular mechanism of *N. chinensis* against AF.

**Methods:**

The TCMSP was used to screen the active *N. chinensis* compounds and their targets. Differentially expressed genes (DEGs) for AF were identified using open-access databases. Using Venn diagrams, the cross-targets of *N. chinensis*, pyroptosis, and AF were obtained. The genes underwent molecular docking as well as gene set enrichment analysis (GSEA). A nomogram based on candidate genes was constructed and evaluated with the clinical impact curve. After that, the immune infiltration of the dataset was analyzed by single sample GSEA (ssGSEA). Finally, microRNAs (miRNAs) and transcription factors (TFs) were predicted based on candidate genes.

**Results:**

Tumor necrosis factor (TNF) and caspase-8 (CASP8) were obtained as candidate genes by taking the intersection of DEGs, targets of *N. chinensis*, and pyroptosis-related genes. Toll-like receptor (TLR) and peroxisome proliferator-activated receptor (PPAR) signaling pathways were linked to candidate genes. Additionally, immune cell infiltration analysis revealed that CASP8 was associated with natural killer T cells, natural killer cells, regulatory T cells (Tregs), myeloid-derived suppressor cells (MDSC), macrophages, CD8 T cells, and CD4 T cells. Finally, miR-34a-5p and several TFs were found to regulate the expression of CASP8 and TNF.

**Conclusion:**

CASP8 and TNF are potential targets of *N. chinensis* intervention in pyroptosis-related AF, and the TLR/NLRP3 signaling pathway may be associated with this process.

## 1 INTRODUCTION

Atrial fibrillation (AF) is a common persistent arrhythmia that affects 33 million people worldwide [1]. Patients with AF are at higher risk of stroke, heart failure, and death than healthy people [2]. The two main types of treatment for AF today are pharmacological, which includes medications to manage heart rate, rhythm, and blood clotting, and surgical, which includes radiofrequency ablation of AF and percutaneous left atrial appendage ligation. Anti-arrhythmic drugs, however, can occasionally be ineffective or have certain side effects [3, 4]. Anticoagulation therapy has difficulty in patient management and bleeding risk control. Although fibrillation activity can be profoundly disrupted by radiofrequency ablation, there is a chance that it will return [5]. Percutaneous left atrial appendage occlusion is not indicated in patients after cardiac surgery [6]. The current prevailing doctrine holds that the onset and maintenance of AF depend on the presence of triggers and substrates, including point and structural remodeling of the atria [7]. In addition, some studies suggest that AF may be associated with extra-atrial pathological changes and is a systemic disease, but the exact mechanism remains to be studied [8]. To date, the mechanisms of AF are not yet fully elucidated, and research on the prevention and treatment of AF still leaves much room to improve.

Regulatory cell death (RCD) is a controlled form of cell death distinct from necrosis, and pyroptosis is one of the most studied RCDs at present [9]. Pyroptosis was first identified by Zychlinsky *et al* in macrophages of *Shigella flexneri*-infected patients [10]. Pyroptosis and apoptosis (the first discovered RCD) have some similarities in their dependence on caspases, so initially, these two could not be well distinguished. However, subsequent studies found that, unlike apoptosis, pyroptosis is a pro-inflammatory RCD [11]. To be specific, pyroptosis is a cellular protective mechanism that inhibits the spread of pathogens by causing cell death but also leads to the development of pathological inflammation in the organism [12]. Caspase-1 is the first identified and most classical inflammatory caspase that induces pyroptosis and is usually activated by inflammasomes generated as a result of bacterial, viral and toxin invasion [13]. Activated caspase-1 cleaves proteins of the gasdermin family and eventually forms pores in the cell membrane, causing pyroptosis [14]. Numerous pathways, including caspase-8 (CASP8) [15] and others, have been gradually discovered in later studies.

Recent evidence suggests that pyroptosis is closely associated with cancer, neurological diseases, autoimmune diseases and cardiovascular diseases [16-19]. Studies have shown that α-synuclein (α-Syn), a characteristic pathological protein of Parkinson's disease (PD), may activate the NLR family pyrin domain containing 3 (NLRP3) inflammasome and induce pyroptosis during the progression of the disease [20, 21]. In contrast, caspase-1 inhibitors inhibit pyroptosis, thereby slowing the progression of PD [22]. In oncological diseases, pyroptosis is considered a way to kill tumor cells by bypassing the apoptosis-inhibiting effect of tumors [23, 24]. But, due to the heterogeneity of oncological diseases, it has been found that pyroptosis can also promote tumor progression [25]. Pyroptosis has also been proven to play an important role in cardiovascular diseases. Inflammatory factors released after pyroptosis are involved in the process of atherosclerosis [26]. In diabetic cardiomyopathy (DCM], pyroptosis is involved in the death of cardiac fibroblasts, leading to cardiac remodeling [27]. Although evidence suggests that inflammation and myocardial fibrosis are closely associated with AF [28], there are no studies on the mechanism of action of pyroptosis in AF.

*Nardostachys chinensis* is a plant of the septoria family whose roots or rhizomes are used in traditional Chinese medicine after drying. In clinical practice, *N. chinensis* is mostly used in herbal combinations for the treatment of AF. To exclude the effect of other herbs, there are also studies on the effect of *N. chinensis* alone on AF or arrhythmias. A study using a post-infarction mouse model showed that mice in the *N. chinensis* group had a lower incidence of ventricular fibrillation and a smaller left ventricular infarct area than those in the blank group [29]. Another study showed that *N. chinensis* has cardioprotective and antiarrhythmic effects [30]. The essential oil of *N. chinensis* has anti-inflammatory and wound healing-promoting effects, which may be related to its inhibition of immune and inflammation-related protein molecules [31]. The effects of *N. chinensis* on AF may also be related to the suppression of immune response and inflammation, but no relevant studies are available.

The multi-component character of herbs poses certain difficulties in the study of their therapeutic mechanisms. Network pharmacology, proposed by Hopkins *et al.* [32], predicts possible mechanisms by screening disease-related targets of components in herbs. Bioinformatics analysis is a type of research based on gene sequencing data. It has been widely used to identify disease-related targets through publicly available data. The combination of bioinformatics and network pharmacology provides a better understanding of the active components and mechanisms of herbs in disease treatment based on computer technology. In this study, combining these two methods, we aimed to explore the mechanisms by which *N. chinensis* inhibits pyroptosis and thus intervenes in AF.

## 2 MATERIALS AND METHODS

### 2.1 Construction of Protein-protein Interaction (PPI) Network

In TCMSP (https://tcmspw.com/tcmsp.php), the keyword “Gan Song” (Chinese for *N. chinensis*) was used to identify oral bioavailability (OB) ≥ 30% and drug similarity (DL) ≥ 0.18 as the selection criteria [33]. The protein sequences of drug targets were normalized to official gene symbols using the UniProt database (https://www.uniprot.org/). The interactions between potential therapeutic targets of *N. chinensis* were obtained through the STRING database (https://www.string-db.org/). The Cytoscape software was used to construct a PPI network to further screen the core therapeutic targets based on the extent.

### 2.2 GEO Data Set Acquisition

Clinical datasets received from the Gene Expression Omnibus (GEO) database (https://www.ncbi.nlm.nih.gov/geo/) with the keywords: (Atrial fibrillation) AND “*Homo sapiens*” (porgn:_txid9606) AND “Expression profiling by array” AND “Series”. GSE31821, GSE41177 and GSE79768 were selected for further study in this study. Among them, GSE31821 contained 4 samples of AF and 2 samples of sinus rhythm, GSE41177 contained 32 samples of AF and 6 samples of sinus rhythm, and the data in GSE79768 contained 14 samples of AF and 12 samples of sinus rhythm. Based on the platform annotation information, this article converted the probes to gene symbols and excluded probes containing multiple genes. In addition, the “sva” package in R was used to remove the batch effect.

### 2.3 Identifications of DEGs

The “limma” package was adopted for identifying the differential expression of DEGs, which was shown in the heatmap through the comparison of the GSE31821, GSE41177 and GSE79768. DEGs with adjusted *p* < 0.05 and |log2 FC| > 2 were considered statistically significant.

### 2.4 Screening and Verification of Candidate Genes

A total of 35 pyroptosis-related genes (PRGs) [34-36] were obtained from previous studies, as shown in Table **S1**. The intersection of PRGs, DEGs and AF-related targets in *N. chinensis* was taken to screen candidate diagnostic genes. Meanwhile, the “ggpubr” package was used to determine the differential expression of candidate genes, and *p* < 0.05 was considered statistically significant. Based on receiver operating characteristic (ROC) curves, the “pROC” package was used to assess the training and test data sets. A two-sided *p* < 0.05 was used to indicate statistical significance.

### 2.5 Functional Enrichment Analyses

GSEA enrichment analyses of candidate genes were performed using the “clusterProfiler” package, and *p* < 0.05 was considered statistically significant.

### 2.6 Construction of the Nomogram

A nomogram was created using candidate genes *via* the “rms” package. Calibration curves were used to assess the accuracy of the nomogram. The clinical utility of the nomogram was assessed by decision curve analysis and clinical impact curves.

### 2.7 Molecular Docking

To predict the binding of candidate gene proteins to small molecules of components of *N. chinensis*, molecular docking was performed after optimizing the molecular structure and the free energy of small ligand molecules and acceptor proteins. First, the PDB files of the target protein were downloaded from the PDB database (http://www.rcsb.org/). The proteins were saved in PDBQT format as the docking acceptor after dehydration and hydrogenation. Next, the small molecule 2D structures were downloaded from the PubChem database (https://pubchem.ncbi.nlm.nih.gov/). The minimum free energy was calculated and the structure was optimized and saved as a mol2 format file. Finally, molecular docking was performed using AutoDock Vina to estimate the binding activity of small molecules to proteins, mainly by the binding free energy. The lower the binding energy value, the greater the likelihood that the ligand will bind to the receptor.

### 2.8 Evaluation of Immune Cell Infiltration

The “GSVA” package was used to screen 28 types of immune cells, and *p* < 0.05 was considered a statistically significant difference. The “pheatmap” package was used to create a heatmap, and the “vioplot” package was used to create a violin map of immune cell expression in patients in the AF and sinus rhythm groups.

### 2.9 Correlation Analysis between Immune Cells and Candidate Genes

The relationship between the infiltrating immune cells and the candidate genes was investigated using Spearman’s rank correlation test in the R software. The chart method provided by the “ggplot2” package was used to display the generated correlations.

### 2.10 Prediction of miRNAs and TFs Engaged with Diagnostic Genes

To further investigate the molecular insight, the transcription factors (TFs) and miRNAs of candidate genes were screened through an online platform. The NetworkAnalyst (https://www.networkanalyst.ca/) platform [37] was applied to find reliable TFs from the ENCODE database that bind to our diagnostic genes. In addition, Tarbase in the NetworkAnalyst platform was used to predict miRNA contacts with candidate genes. Interaction networks of diagnostic genes with TFs and miRNAs were created in Cytoscape.

## 3 RESULTS

The flow chart of our study is shown in Fig. (**1**).

### 3.1 Protein-protein Interaction (PPI) Network

After setting the interaction score to 0.9 and hiding the isolated nodes, we screened the 439 targets obtained from the STRING database. The results were exported as TSV format files and visualized with Cytoscape software. The PPI network consisted of 319 nodes and 1299 edges. The CytoNCA plug-in in Cytoscape was used for topological analysis and the target genes that simultaneously met the values of each parameter greater than the median value were combined and de-weighted with the following filtering conditions: betweenness centrality (BC) = 104.13, closeness centrality (CC) = 0.07, degree centrality (DC) = 5, eigenvector centrality (EC) = 0.01, local average connectivity-based method (LAC) = 1.78, and network centrality = 2.75. The results are shown in Fig. (**2a** and **b**) and Table **S2**, and a total of 82 key genes were obtained.

### 3.2 GEO Data Set DEGs Screening

**Fig. (3):**
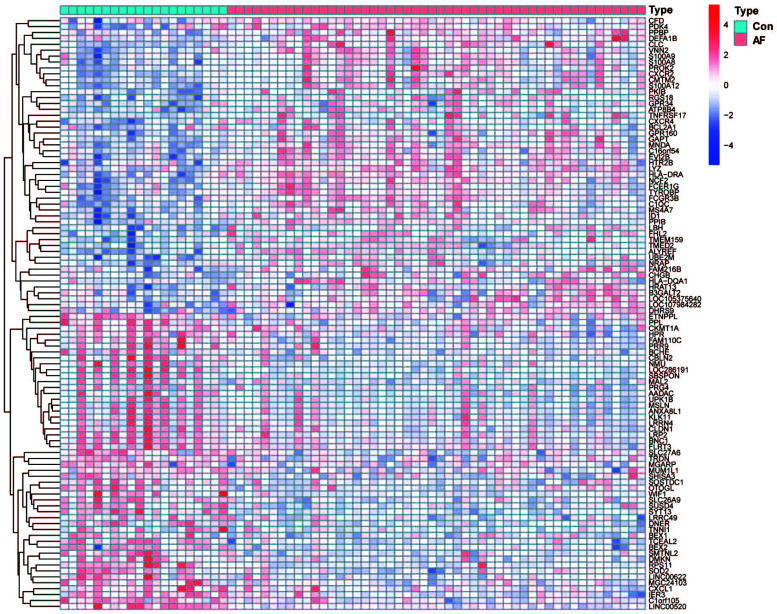
Heatmap of DEGs between AF and controls in datasets GSE31821, GSE41177, and GSE79768. Red: upregulated; blue: downregulated.

2137 DEGs were identified between patients with AF and controls (Table **S3**), and the heatmap showed the expression of DEGs between the two groups (Fig. **3**).

### 3.3 Screening and Verification of Candidate Genes

**Fig. (4):**
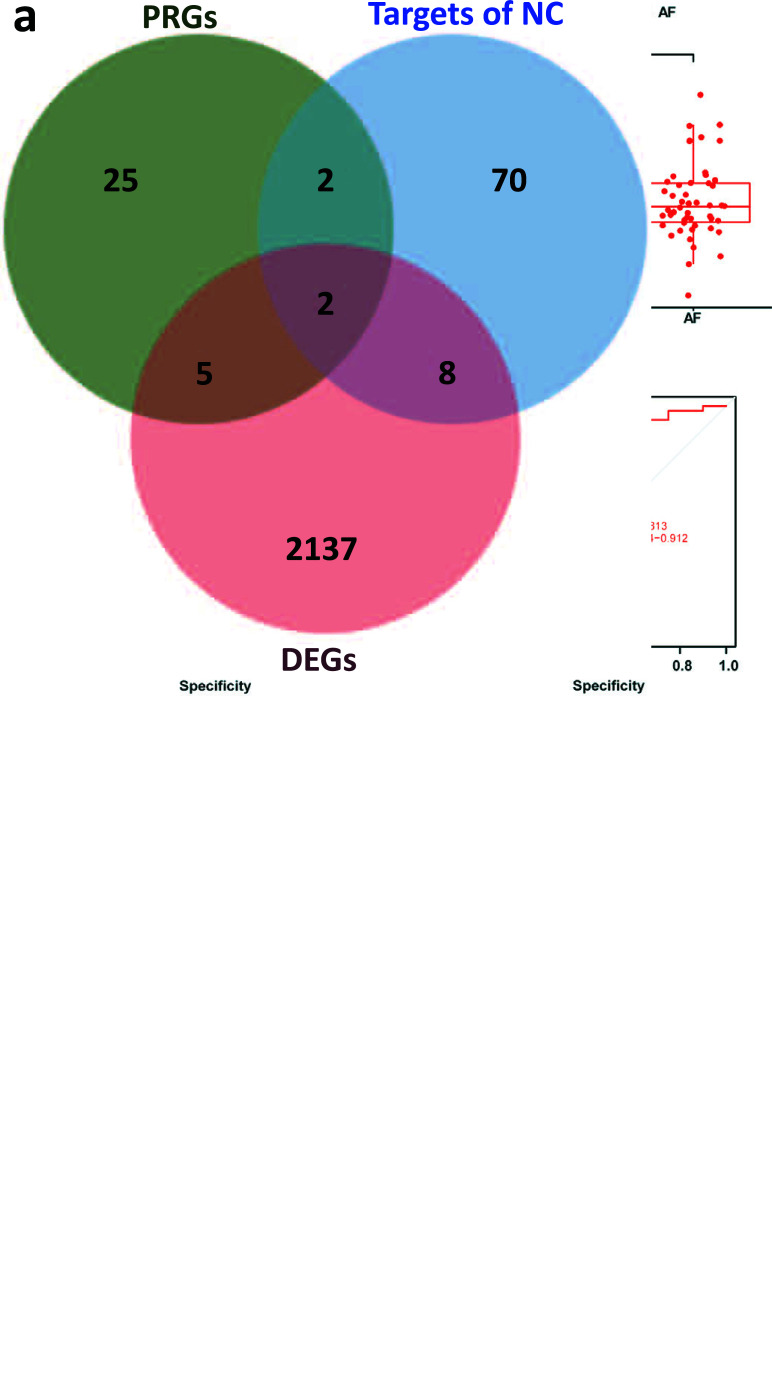
Candidate genes screening. (**a**) The Venn diagram of the potential therapeutic targets of *N. chinensis*, PRGs, and DEGs. (**b**) The boxplot of CASP8 expression in patients with AF (red) and sinus rhythm (blue). (**c**) The boxplot of TNF expression in patients with AF (red) and sinus rhythm (blue). (**d**) The ROC curve of CSAP8. (**e**) The ROC curve of TNF. (***: *p* < 0.001).

Intersections were taken for *N. chinensis*-related targets (82 genes), DEGs (2137 genes), and PRGs (35 genes). Tumor necrosis factor (TNF) and caspase-8 (CASP8) were screened as candidate genes (Fig. **4a**). Visualizing the expression of TNF and CASP8 by box plots (Fig. **4b** and **c**), the expression of CASP8 (*p* < 0.001) was upregulated in patients with AF and the expression of TNF (*p* < 0.001) was downregulated in patients with AF. ROC curves were generated for CASP8 and TNF to validate the diagnostic ability, with an area under the curve of 0.824 and 0.813 in the training dataset, respectively (Fig. **4d** and **e**). The area under the curves was used to determine their effectiveness in distinguishing AF patients from healthy controls, which means that both CASP8 and TNF have good efficacy in the diagnosis of AF.

### 3.4 Enrichment Analyses

**Fig. (5):**
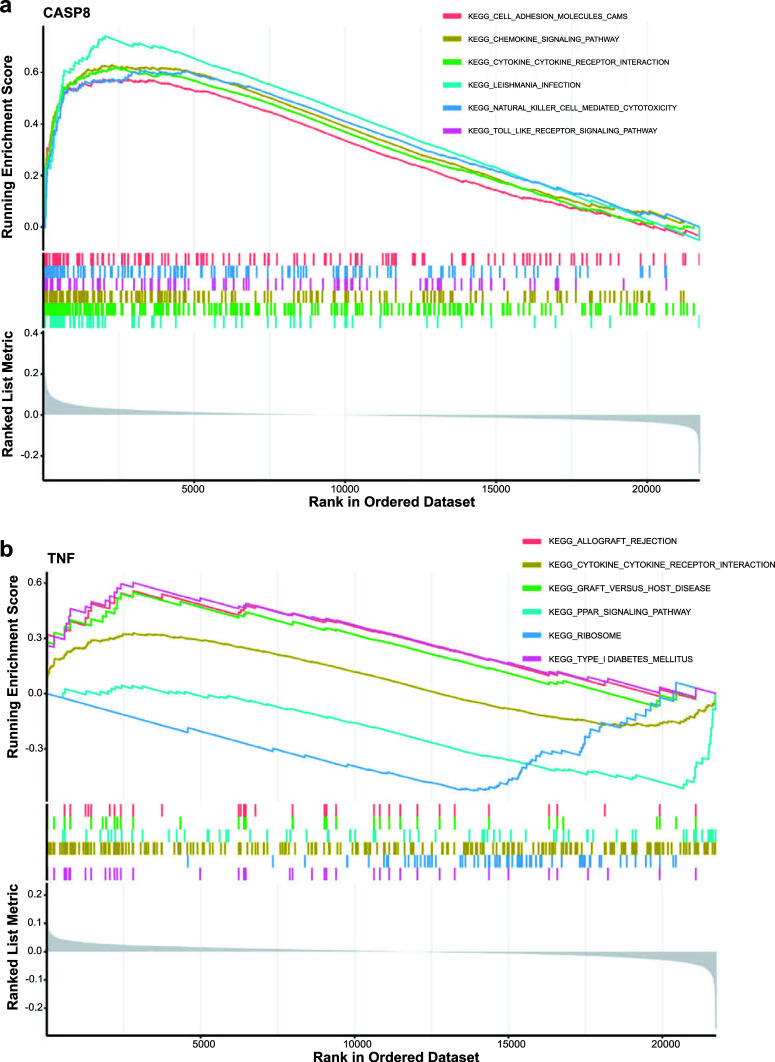
GSEA of two candidate genes. (**a**) GSEA of CASP8. (**b**) GSEA of TNF.

GSEA revealed that candidate genes were closely associated with natural killer cell-mediated cytotoxicity, cytokine-cytokine receptor interaction, chemokine signaling pathway, toll-like receptor (TLR) signaling pathway and peroxisome proliferator-activated receptor (PPAR) signaling pathway (Fig. **5a** and **b**).

### 3.5 Nomogram of Diagnostic Genes

**Fig. (6):**
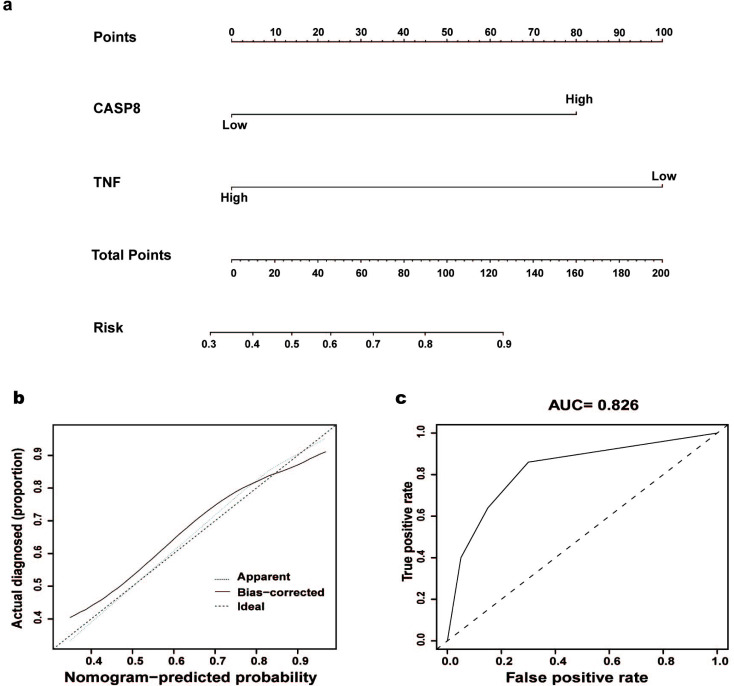
Diagnostic gene-based nomogram for predicting AF. (**a**) A nomogram predicting the risk of CASP8 and TNF for patients with AF. (**b**) The calibration curves for the nomogram. The X-axis represents the nomogram-predicted probability. The Y-axis represents the actual probability of invasive adenocarcinoma. (**c**) The clinical impact curve for the nomogram. (AUC = 0.826).

We then constructed a nomogram as a tool for diagnosing AF (Fig. **6a**) using candidate genes. In the nomogram, the expression levels of the two diagnostic genes together constitute the AF risk score. The calibration curve showed that the nomogram was able to assess the progression of AF (Fig. **6b**) accurately. The clinical validity of the nomogram was demonstrated by the clinical impact curve (Fig. **6c**). According to the results above, we found that high expression of CASP8 and low expression of TNF may raise the risk of AF.

### 3.6 Molecular Docking

**Fig. (7):**
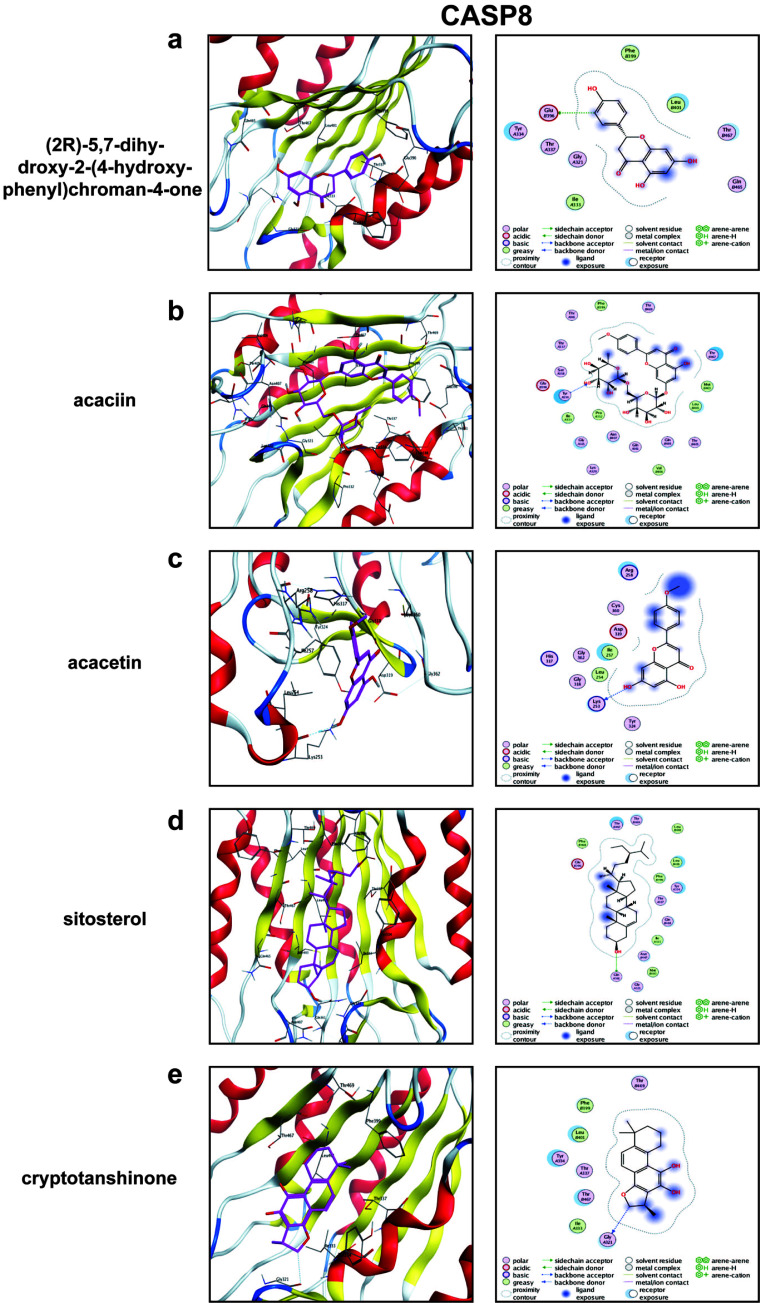
Molecular docking results. (**a**-**e**) The action mode of CASP8 with each of the 5 components. Yellow dashed lines indicate hydrogen bonds, solid-colored structures indicate binding sites, and heterochromatic structures indicate drug components.

**Fig. (8):**
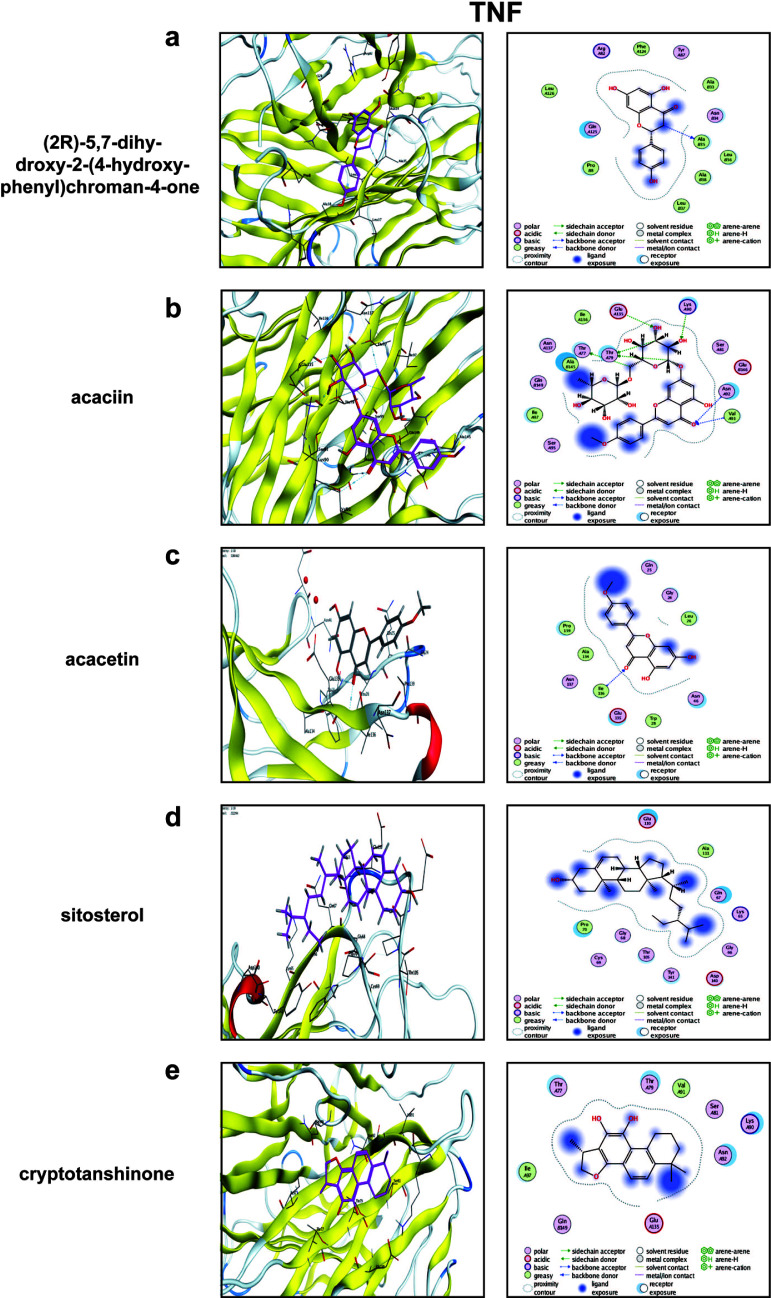
Molecular docking results. (**a**-**e**) The action mode of TNF with each of the 5 components. Yellow dashed lines indicate hydrogen bonds, solid-colored structures indicate binding sites, and heterochromatic structures indicate drug components.

We docked each of the five small molecules and the two core targets, and the results showed that all had good binding power (Table **1**). The protein PDB IDs of CASP8 and TNF collected from the database were 6X8H and 1A8M, respectively. We found that CASP8 had a minimum binding energy of -8.9 kcal-mol-1, while TNF had a minimum binding energy of -8.0 kcal-mol-1, indicating that sitosterol and acacetin are the key components that play a regulatory role. The results are shown in Figs. (**7** and **8**).

(2R)-5,7-dihydroxy-2-(4-hydroxyphenyl)chroman-4-one formed a hydrogen bond with the CASP8 protein at the amino acid residue Tyr 334 (Fig. **7a**). Acaciin formed hydrogen bonds with the CASP protein at amino acid residues Tyr 334 and Ile 333 (Fig. **7b**). Acacetin was linked to Lys 253, Arg 258, and Asp 319 of CASP8 protein *via* hydrogen bonds (Fig. **7c**). Sitosterol and CASP8 protein were also hydrogen-bonded at amino acid residues Gln 361 (Fig. **7d**). Tyr 334 of CASP8 formed a hydrogen bond with cryptotanshinone (Fig. **7e**).

(2R)-5,7-dihydroxy-2-(4-hydroxyphenyl)chroman-4-one formed three pairs of hydrogen bonds with the TNF protein at amino acid residues Tyr 87, Asn 34, and Gln 125 (Fig. **8a**). Acaciin was linked to the TNF protein by hydrogen bonds to Lys 90, Glu 135, Asn 92, and Gln 149 (Fig. **8b**). The TNF protein formed a hydrogen bond with acaciin at Ser 99 (Fig. **8c**).

### 3.7 Infiltration of Immune Cells Results and Correlation with Key Biomarkers

**Fig. (9):**
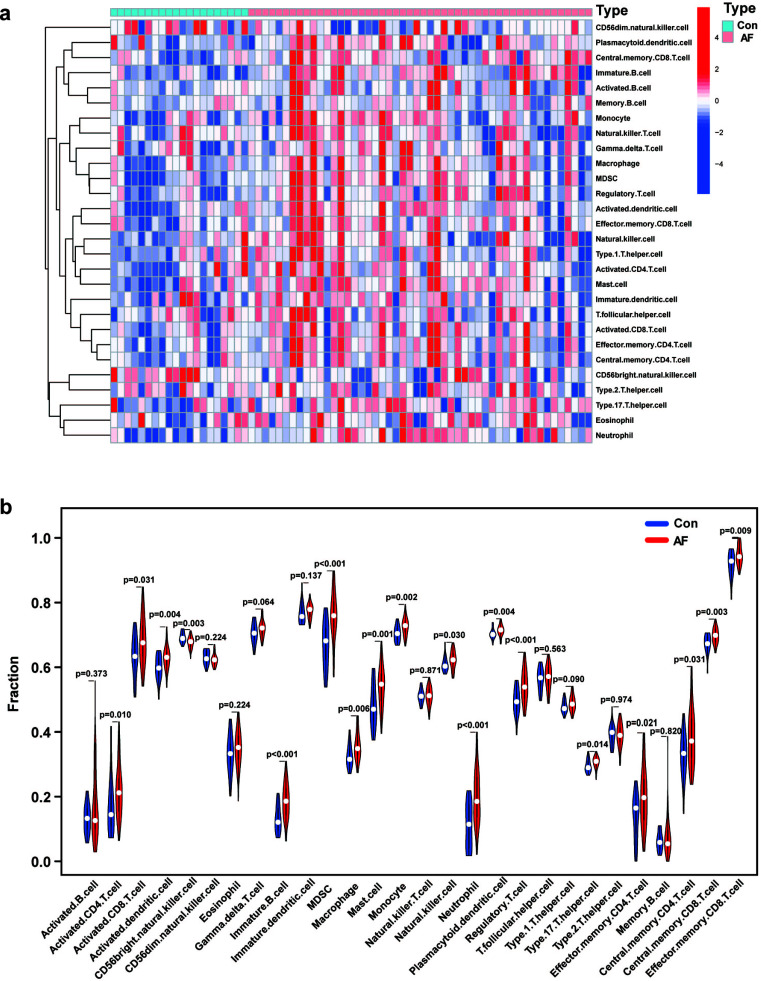
Immune cell infiltration in AF and sinus rhythm tissues. (**a**) The expression of 28 immune cells in each sample. Red: upregulated; blue: downregulated. (**b**) The differences in expression of each of the 28 immune cells between patients with AF and sinus rhythm. Red: patients with AF, blue: patients with sinus rhythm.

Using the ssGSEA algorithm, we scored the expression of 28 immune cells. We visualized the results using a heatmap (Fig. **9a**). As shown in the violin plot, the expression of a total of 18 immune cells differed between patients with sinus rhythm and AF (Fig. **9b**). Expression of activated CD4 T cell (*p* = 0.010), activated CD8 T cell (*p* = 0.031), activated dendritic cell (*p* = 0.004), immature B cell (*p* < 0.001), myeloid-derived suppressor cell (MDSC) (*p* < 0.001), macrophage (*p* = 0.006), mast cell (*p* = 0.001), monocyte (*p* = 0.002), natural killer cell (*p* = 0.030), neutrophil (*p* < 0.001), plasmacytoid dendritic cell (*p* = 0.004), regulatory T cell (*p* < 0.001), type 17 T helper cell (*p* = 0.014), effector memory CD4 T cell (*p* = 0.021), central memory CD4 T cell (*p* = 0.031), central memory CD8 T cell (*p* = 0.003) and effector memory CD8 T cell (*p* = 0.009) were upregulated compared to patients with sinus rhythm. Expression of CD56 bright natural killer cell (*p* = 0.003) in patients with AF was downregulated compared to patients in sinus rhythm.

### 3.8 Correlations Between Diagnostic Genes and Immune Cells

The results of the correlation analysis shown in Fig. (**10**) indicated that CASP8 was positively correlated with type 2 T helper cell (*p* < 0.01), type 17 helper cell (*p* < 0.05), type 1 helper cell (*p* < 0.001), T follicular helper cell (*p* < 0.001), natural killer T cell (*p* < 0.001), natural killer cell (*p* < 0.001), monocyte (*p* < 0.01), memory B cell (*p* < 0.01), MDSC (*p* < 0.001), mast cell (*p* < 0.01), macrophage (P < 0.001), immature dendritic cell (*p* < 0.01), immature B cell (*p* < 0.001), gamma delta T cell (*p* < 0.01), effector memory CD8 T cell (*p* < 0.001), central memory CD4 T cell (*p* < 0.001), activated dendritic cell (*p* < 0.01), activated CD8 T cell (*p* < 0.001), activated CD4 T cell (*p* < 0.01) and activated B cell (*p* < 0.01). TNF was positively correlated with immature B cell (*p* < 0.05) and negatively correlated with type 2 T helper cell (*p* < 0.05), immature dendritic cell (*p* < 0.05) and CD56 bright natural killer cell (*p* < 0.05).

### 3.9 Prediction of miRNA and TFs

**Fig. (11):**
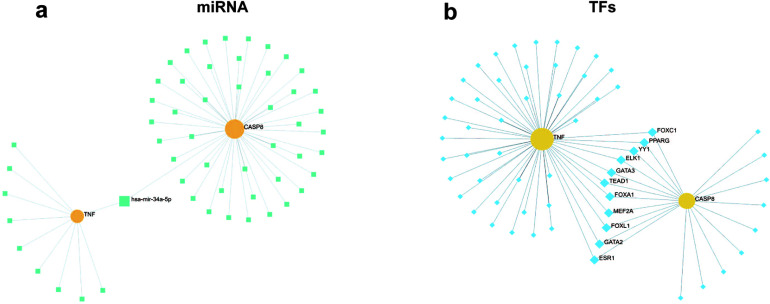
Prediction of miRNA and TFs. (**a**) Common miRNA of CASP8 and TNF (light green square). (**b**) Common TFs of CASP8 and TNF (light blue squares).

The prediction results showed that miR-34a-5p may be the common miRNA of TNF and CASP8 (Fig. **11a**), and FOXC1, PPARG, YY1, ELK1, GATA3, TEAD1, FOXA1, MEF2A, FOXL1, GATA2, and ESR1 may be the common transcription factors of TNF and CASP8 (Fig. **11b**).

## 4 DISCUSSION

The incidence of AF is increasing year by year, and the majority of patients with AF have an abrupt onset. The current clinical treatment of AF is still somewhat inadequate [38]. A study has shown that Chinese medicines combined with antiarrhythmic drugs have better efficacy and fewer adverse effects, which provides a new direction for the research of AF [39]. Several studies have shown that herbal compounds containing *N. chinensis* can effectively intervene in AF by inhibiting atrial fibrosis, blocking atrial sodium channels, alleviating metabolic syndrome, and reducing P-wave dispersion [40-43], but the role that *N. chinensis* plays in this process is unclear.

After screening the candidate genes, we finally obtained two genes, TNF and CASP8. The clinical prediction model constructed with these two genes as variables also performed well in this study. CASP8 is a member of the cysteine protease family, it is associated with biological processes such as anoikis and autophagy [44, 45]. More importantly, CASP8 is also a key hub for three forms of RCD: apoptosis, pyroptosis and necroptosis [46]. Typically, CASP8 is the promoter of exogenous apoptosis [47], it is also involved in the cleavage of RIPK1 and RIPK3, thereby inhibiting necroptosis [48]. *Yersinia* effector protein YopJ was found to induce CASP8-related GSDMD cleavage in macrophages, ultimately leading to pyroptosis [49]. Chemotherapeutic drugs were found to mediate tumor cell pyroptosis *via* CASP8 in breast cancer cells [23]. In an observational study, the expression level of CASP8 was positively associated with the risk of recurrence of AF after radiofrequency ablation [50]. The use of the anti-inflammatory molecule resolvin-D1 resulted in lower CASP8 levels and less right atrial fibrosis in mice in the intervention group than in mice with right heart disease [51]. In our study, the expression of CASP8 was upregulated in patients with AF compared to controls, which is consistent with the current studies.

TNF, fully known as tumor necrosis factor, has an important role in the homeostasis of the body and the development of several diseases [52]. TNF can cause caspase cascade activation and induce apoptosis by assembling into complexes in the cytoplasm [53]. It has also been found that TNF-α can induce cleavage of GSDMC *via* CASP8, triggering pyroptosis [25]. Studies have shown that elevated levels of TNF-α can promote atrial fibrosis and atrial remodeling, thereby inducing AF [54, 55]. An animal experiment showed that inhibition of the expression of TNF reduced the occurrence of AF after myocardial infarction [56]. In our study, TNF was shown to be downregulated in patients with AF, contrary to the above findings, but TNF performed well in subsequent diagnostic validity tests. GSEA showed that the downregulation of TNF correlated with upregulation of the PPAR signaling pathway. Several studies have shown that PPAR can reduce the occurrence of pyroptosis by inhibiting NLRP3 and NF-κB signaling pathways [57-59]. Thus, we suggested that this may be a balancing mechanism within the organism. Therefore, we suggest that pyroptosis may be involved in the progression of AF, and *N. chinensis* may intervene in such a process through CASP8 and TNF. To further clarify the components of *N. chinensis* that play a role in it, we conducted a follow-up study based on molecular docking.

Molecular docking can predict ligand-target interactions at the molecular level, thus helping us to screen for therapeutically significant herbal ingredients [60]. The main components of *N. chinensis* are volatile oils and terpenoids [61]. In this study, we obtained five major bioactive ingredients, (2R) -5,7-dihydroxy-2-(4-hydroxyphenyl)chroman-4-one, acaciin, acacetin, sitosterol, and cryptotanshinone. In molecular docking, acacetin and sitoterol bind more strongly to two hub genes. Acacetin has been shown in an animal study to be an atrium-selective inhibitor of outward K^+^ current and to be effective in preventing AF [62]. A study showed that acacetin could reduce the expression of NLRP3 and TNF-α to lessen ischemia-reperfusion injury after cerebral infarction [63]. The sitosterol may exert anti-inflammatory effects by inhibiting the transcriptional activity of NF-κB [64], and studies have shown that activation of NF-κB promotes the activation of NLRP3 inflammasome [65]. Cryptotanshinone specifically inhibits the activity of NLRP3 inflammasome and is a potential drug for the treatment of NLRP3 inflammasome-mediated diseases [66]. Acaciin, also known as linear, has been shown to reduce the release of inflammatory factors and inhibit the NLRP3 signaling pathway to improve acute lung injury [67]. NLRP3 can induce pyroptosis [68], and there have been a number of studies of NLRP3 signaling pathways mediating AF in recent years [69-71]. In GSEA, the CASP8 was associated with the toll-like receptor (TLR) signaling pathway, which has been shown to be associated with the formation of NLRP3 inflammasome [72]. A network pharmacology study suggests that (2R)-5,7-dihydroxy-2-(4-hydroxyphenyl)chroman-4-one may be involved in regulating inflammatory factor levels and alleviating diabetic retinopathy [73]. Taken together, we suggest that *N. chinensis* may ameliorate AF mainly by inhibiting the TLR/NLRP3 signaling pathway-mediated pyroptosis. In recent years, the pathway of CASP8-induced NLRP3 activation has been gradually discovered, and the inflammatory response cuts through this process [74, 75]. Given the activation and chemotaxis of immune cells are closely related to the inflammatory response [76], we performed an immune infiltration analysis to explore the role played by immune cells in the process of AF.

We used ssGSEA to determine the differences in the expression of 28 different types of immune cells in patients with AF and control. The results showed that a total of 18 immune cells differed between the two groups, except for CD56-bright natural killer (NK) cells. The expression levels of the remaining 17 immune cells were higher in AF patient species. Cd56 bright NK cells are an important subpopulation of NK cells, and their role is currently unclear. It has been shown that CD56 bright NK cells can produce large amounts of the immunosuppressive factor IL-10 to downregulate immune responses [77], and our results suggest that the immunomodulatory role of CD56 bright NK cells is suppressed in the development of AF. Of the two key genes screened, CASP8 showed a stronger correlation with the expression of immune cells. Among the immune cells that were highly and positively correlated with CASP8, those highly expressed in the patients with AF were CD8 T cells, CD4 T cells, regulatory T cells (Tregs), myeloid-derived suppressor cell (MDSC), macrophages, plasmacytoid dendritic cells, NK cells, and NK T cells. It was shown that high expression of GSDME, which causes pyroptosis, enhances the number and function of CD8 T cells in tumor cells [78]. Upregulated CD8 T cell expression was also found in patients with postoperative atrial fibrillation (POAF) [79], and another study found that loss of CD28 antigen in CD4 T cells may be a potential mechanism of AF [80]. Plasmacytoid dendritic cells are a subset of dendritic cells, and both dendritic cells and macrophages are derived from MDSC. In a study of sepsis-associated acute lung injury, the exosome TNF-Exo promotes M1-type macrophage polarization while also initiating macrophage pyroptosis [81]. It has been shown that macrophage M1 polarization in AF further exacerbates atrial electrical remodeling [82]. Tregs and NKT cells have immunomodulatory properties. Luteolin was found to promote IL-10 secretion by Tregs to reduce pyroptosis in lung cells [83], but Tregs have also been reported to promote pyroptosis and increase lung injury in Aspergillus fumigatus infection mice [84]. However, the Tregs have only been found to attenuate the inflammatory response and reduce atrial fibrosis in AF [85, 86]. NKT cells can regulate both innate and adaptive immunity, and its pathological mechanisms in pyroptosis and AF are unclear. Studies have shown that both acacetin and cryptotanshinone inhibit T cell activation and proliferation [87, 88], and acacetin also regulates the differentiation of Tregs [89]. Acacetin and acaciin inhibit the inflammatory response of macrophages [90, 91], and cryptotanshinone inhibits macrophage migration [92]. Therefore, *N. chinensis* is also related to its regulation of immune cells when intervening in pyroptosis-associated AF.

In the end, we used a network-based approach [37] and found that miR-34a-5p may be the most important miRNA. A clinical study found that miR-34a-5p expression among outpatients with AF was upregulated in both the 12-month follow-up group and the 24-month follow-up group and miR-34a-5p regulated the largest number of target genes [93]. Another study suggested that miR-34a-5p may play a role in AF by regulating the expression of Ankyrin-B (ANk-B) [94]. Studies have shown that miR-34a-5p acts as a miRNA for caspase-1 to induce pyroptosis [95]. Therefore, miR-34a-5p has great potential to be an upstream target for intervention in AF through pyroptosis.

There are still limitations in this study due to the lack of validation in clinical or animal studies. Future research will combine molecular biology and pathophysiology to validate the predicted potential key targets and pathways.

## CONCLUSION

Our study found that CASP8 and TNF may be the targets of *N. chinensis* in the treatment of pyroptosis-mediated AF. The active compounds of *N. chinensis* may intervene in AF by modulating the TLR/NLRP3 signaling pathway and macrophages, thereby reducing the level of inflammation in the atria and decreasing the occurrence of pyroptosis. Meanwhile, miR-34a-5p and several TFs, including FOXC1, PPARG, YY1 can regulate the expression of CASP8 and TNF, which can also be used as targets for drug intervention in AF.

## Figures and Tables

**Fig. (1):**
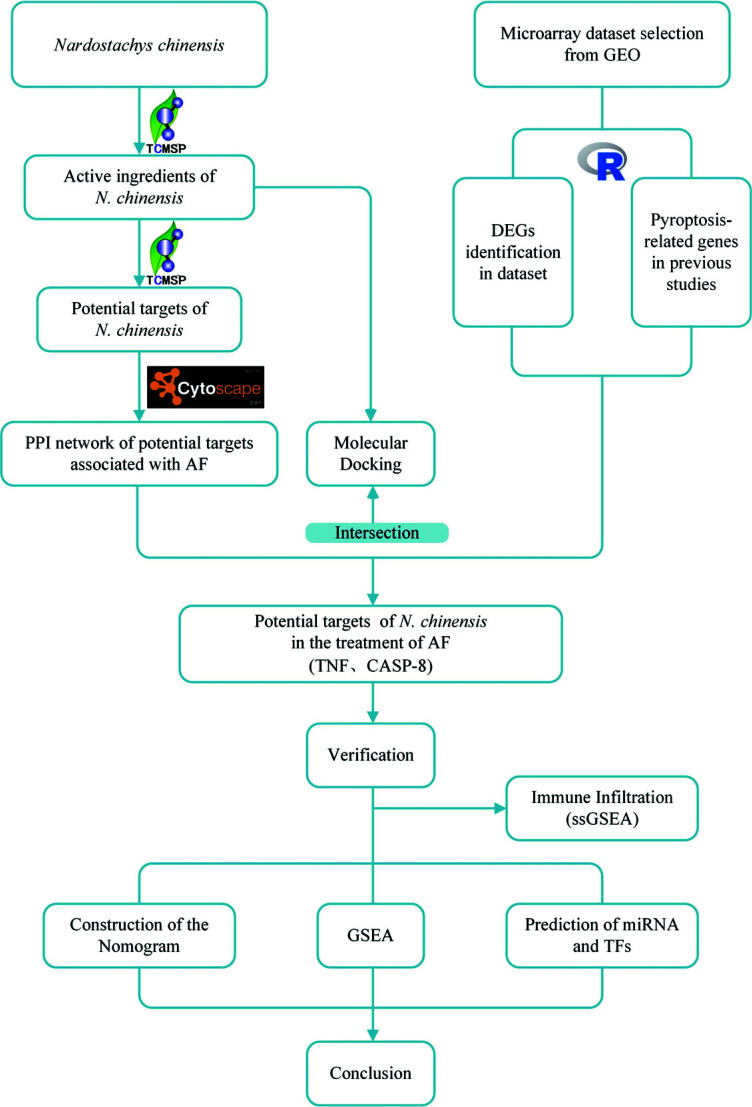
Flow chart of the study.

**Fig. (2):**
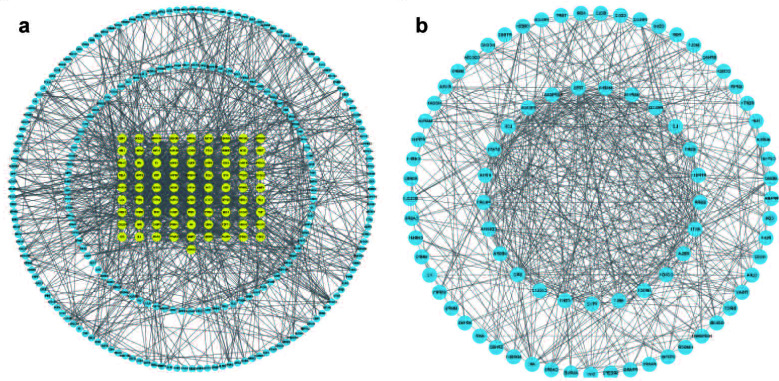
**(A** and **B)** The network of the relationship between the active ingredients and the targets of *N. chinensis*.

**Fig. (10):**
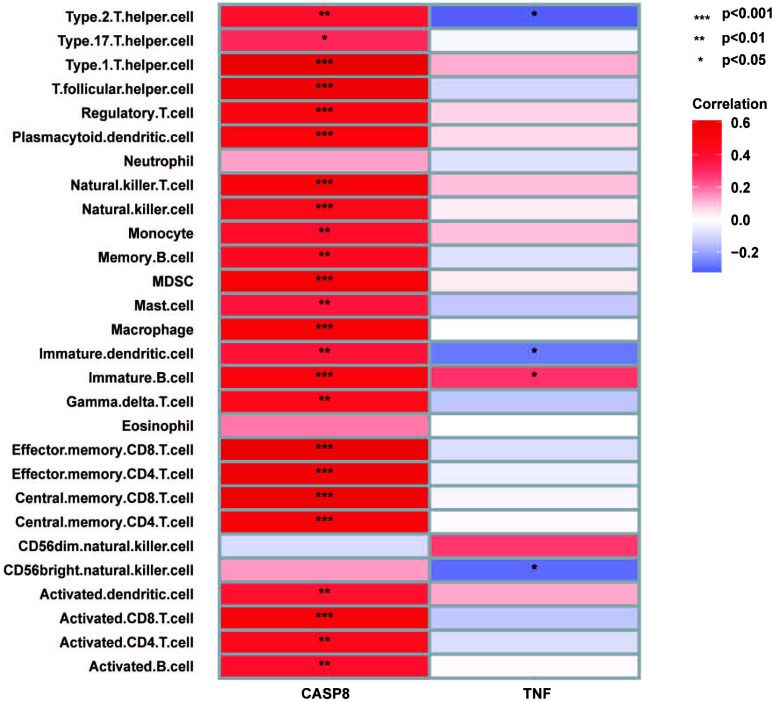
Correlation between biomarkers and differential immune cells in AF.

**Table 1:** The binding energy of active components and key genes.

**Component**	**Gene**	**PDB ID**	**Binding Energy [kcal·mol-1]**
(2R)-5,7-dihydroxy-2-(4-hydroxyphenyl)chroman-4-one	CASP8	6X8H	-6.8
Acaciin	CASP8	6X8H	-8.1
Acacetin	CASP8	6X8H	-7.3
Sitosterol	CASP8	6X8H	-8.9
Cryptotanshinone	CASP8	6X8H	-8.7
(2R)-5,7-dihydroxy-2-(4-hydroxyphenyl)chroman-4-one	TNF	1A8M	-6.5
Acaciin	TNF	1A8M	-7.5
Acacetin	TNF	1A8M	-8.0
Sitosterol	TNF	1A8M	-6.1
Cryptotanshinone	TNF	1A8M	-6.7

## Data Availability

The data sets presented in this study can be found in online repositories. The names of the repository/repositories and accession number(s) can be found in the article/Supplementary Material.

## LIST OF ABBREVIATIONS

AF: Atrial Fibrillation
BC: Betweenness Centrality
CASP8: Caspase-8
CC: Closeness Centrality
DC: Degree Centrality
DCM: Diabetic Cardiomyopathy
DEGs: Differentially Expressed Genes
EC: Eigenvector Centrality
GEO: Gene Expression Omnibus
GSEA: Gene Set Enrichment Analysis
NLRP3: NLR Family Pyrin Domain Containing 3
PPAR: Peroxisome Proliferator-activated Receptor
RCD: Regulatory Cell Death
TFs: Transcription Factors
TNF: Tumor Necrosis Factor
