# Supplementary material for: Network Pharmacology and Bioinformatics Analyses Identify the Core Genes and Pyroptosis-related Mechanisms of Nardostachys chinensis for Atrial Fibrillation
Source: Curr Comput Aided Drug Des. 2024 Jan 3;20(7):1070–86. doi: 10.2174/0115734099259071231115072421 (PMC11475257; doi:10.2174/0115734099259071231115072421)
Supplement: Supplementary file 1 [file CCADD-20-1070_SD1.pdf]

SUPPLEMENTARY MATERIAL

Network Pharmacology and Bioinformatics Analyses Identify the Core Genes and Pyroptosis-Related Mechanisms of *Nardostachys Chinensis* for Atrial Fibrillation

Weiqi Xue[1], Yuan Luo[1], Weifeng He[1], Mengyuan Yan[1], Huanyi Zhao[2,*] and Lijin Qing[2,*]

[1]*First School of Clinical Medicine, Guangzhou University of Chinese Medicine, Guangzhou, China;* [2]*First Affiliated Hospital of Guangzhou University of Chinese Medicine, Guangzhou, China*

Supplementary Table 1. 35 pyroptosis-related genes

Genes	Full-names
BAK1	BCL2 antagonist/killer 1
BAX	BCL2 associated X, apoptosis regulator
CASP1	cysteine-aspartic acid protease-1
CASP3	cysteine-aspartic acid protease-3
CASP4	cysteine-aspartic acid protease-4
CASP5	cysteine-aspartic acid protease-5
CHMP2A	charged multivesicular body protein 2A
CHMP2B	charged multivesicular body protein 2B
CHMP3	charged multivesicular body protein 3
CHMP4A	charged multivesicular body protein 4A
CHMP4B	charged multivesicular body protein 4B
CHMP4C	charged multivesicular body protein 4C
CHMP6	charged multivesicular body protein 6
CHMP7	charged multivesicular body protein 7
GSDMD	gasdermin D
GSDME	gasdermin E
HMGB1	high mobility group box-1
IL18	interleukin 18
IL1A	interleukin 1 alpha
IL1B	interleukin 1 beta
TP53	tumor protein p53
CASP6	cysteine-aspartic acid protease-6
CASP8	cysteine-aspartic acid protease-8
CASP9	cysteine-aspartic acid protease-9
GPX4	glutathione peroxidase 4
GSDMB	gasdermin B
GSDMC	gasdermin C
IL6	interleukin 6
NLRP1	NLR family pyrin domain containing 1
NLRP3	NLR family pyrin domain containing 3
NLRP6	NLR family pyrin domain containing 6

NLRP7	NLR family pyrin domain containing 7
NOD1	nucleotide binding oligomerization domain containing 1
NOD2	nucleotide binding oligomerization domain containing 2
TNF	tumor necrosis factor
